# Social Interaction With an Anonymous Opponent Requires Increased Involvement of the Theory of Mind Neural System: An fMRI Study

**DOI:** 10.3389/fnbeh.2022.807599

**Published:** 2022-05-13

**Authors:** Maya Zheltyakova, Alexander Korotkov, Ruslan Masharipov, Artem Myznikov, Michael Didur, Denis Cherednichenko, Lisa Wagels, Ute Habel, Maxim Kireev, Mikhail Votinov

**Affiliations:** ^1^N.P. Bechtereva Institute of the Human Brain, Russian Academy of Science, Saint Petersburg, Russia; ^2^Department of Psychiatry, Psychotherapy and Psychosomatics, Medical Faculty, RWTH Aachen University, Aachen, Germany; ^3^Institute for Cognitive Studies, Saint Petersburg State University, Saint Petersburg, Russia

**Keywords:** anonymity, theory of mind, competitive game, functional connectivity, fMRI

## Abstract

An anonymous interaction might facilitate provoking behavior and modify the engagement of theory of mind (TOM) brain mechanisms. However, the effect of anonymity when processing unfair behavior of an opponent remains largely unknown. The current functional magnetic resonance imaging (fMRI) study applied the Taylor aggression paradigm, introducing an anonymous opponent to this task. Thirty-nine healthy right-handed subjects were included in the statistical analysis (13 males/26 females, mean age 24.5 ± 3.6 years). A player winning the reaction-time game could subtract money from the opponent during the task. Participants behaved similarly to both introduced and anonymous opponents. However, when an anonymous opponent (when compared to the introduced opponent) subtracted money, the right inferior frontal gyrus (IFG) demonstrated an increased BOLD signal and increased functional connectivity with the left IFG. Further, increased functional connectivity between the right IFG, the right temporal parietal junction and precuneus was observed during the perception of high provocation (subtracting a large amount of money) from the anonymous compared to the introduced opponent. We speculate that the neural changes may underlie different inferences about the opponents’ mental states. The idea that this reorganization of the TOM network reflects the attempt to understand the opponent by “completing” socially relevant details requires further investigation.

## Introduction

Currently, an increasing number of social interactions occur online. Apart from the positive aspects, an online environment provides conditions for aggressive behavior (Demsar et al., 2021), which might be because most virtual communication is anonymous (Pornari and Wood, 2010; Runions and Bak, 2015). The term “aggression” can refer to “any behavior directed toward another individual that is carried out with the intent to cause harm” (Anderson and Bushman, 2002). Anonymity is a factor that influences social behavior, such that it can be characterized by higher levels of aggression (Zimmerman and Ybarra, 2016), a greater tendency to punish unfairness (Yu et al., 2015), and an increased self-interest in economic games (Burnham, 2003; Charness and Gneezy, 2008) compared to conditions when opponents know each other’s true identity. An important facet of aggressive behavior in an anonymous online environment is understanding another person’s mental states, especially in the case of receiving provocation. The ability to attribute thoughts, beliefs, and intentions to oneself and others is paraphrased as the theory of mind (TOM) (Premack and Woodruff, 1978), which is underpinned by a set of brain regions, the TOM network (Schurz et al., 2020a). According to meta-analytic studies, the network mainly includes the bilateral temporoparietal junction (TPJ), the medial prefrontal cortex (mPFC), the precuneus, and the superior temporal sulcus (Mar, 2011; Bzdok et al., 2012; Dufour et al., 2013; Molenberghs et al., 2016). Additional parts of the TOM network comprise the temporal pole, the middle temporal gyrus, the right visual cortex (MT/V5), the amygdala, and the inferior frontal gyrus (IFG) (Mar, 2011; Bzdok et al., 2012). The neural basis of TOM abilities has been extensively studied in both normal and pathological states [e.g., autism spectrum disorders (Andreou and Skrimpa, 2020), borderline personality disorder (Bora, 2021), psychopathy (van Dongen, 2020), schizophrenia (Vucurovic et al., 2020)]. However, data regarding the re-organization of the TOM network in response to social provocation in anonymous competition contexts are lacking to our knowledge.

Thus, we aimed to study local activity and connectivity of the TOM network during the interaction with an anonymous repeatedly provoking opponent. We base our hypotheses on the assumption that increased local activity in one given area will reflect increased involvement in processes underlying such an interaction. Increased functional connectivity is assumed to support the mental and behavioral processes during the interaction via a stronger communication between the involved structures. The opposite patterns are assumed to indicate reduced involvement in the processes underlying the interaction in the TAP. To address this question, we applied a functional magnetic resonance imaging (fMRI)-compatible version of the Taylor aggression paradigm (TAP) (Repple et al., 2017; Wagels et al., 2019). In this paradigm, participants select a monetary punishment in response to prior social provocation by the opponent. TOM areas are associated with aggressive decision-making during the TAP (Beyer et al., 2014b), but modifying the type of experimental task and considering individual differences between subjects may provide a better understanding of the role of the TOM network. For example, in a previous version of TAP where participants had an alternative to avoid aggression, the activity in the IFG and the TPJ was decreased during aggressive responses compared to avoiding aggression (Buades-Rotger et al., 2017). In addition, participants with high emotional reactivity to threat, measured as fear potentiation (FP) of the startle response, had lower activity in the mPFC, TPJ, precuneus, and IFG during punishment selection for a highly provoking opponent (Beyer et al., 2014a). Different activity might be related to differences in the interpretation of the opponent’s action. Such an interpretation might differ depending on how much a participant knows about the opponent. In order to manipulate this knowledge about the opponent, we modified the TAP task as follows (see ‘Stimuli and procedure’ in the Materials and Methods section for more details): (1) The participants were told they would interact with two different opponents. While they were introduced to one opponent during the instruction (the known opponent), they did not meet the other opponent prior to or after the experiment (the anonymous opponent). (2) We separated the decision-making phase (selecting the punishment) from the motor performance of this action.

The first evidence characterizing brain mechanisms underlying the interaction with an opponent whose identity is unknown or uncertain (anonymous opponent), compared to an interaction with a person with a known identity (known opponent), is provided by electrophysiological studies investigating event-related potentials (ERPs) using an ultimatum game and dictator game (Wu et al., 2011; Yu et al., 2015). These games required the participants to evaluate fair and unfair monetary distributions decided by the opponent. Fairness evaluation is important to establish cooperative behavior, however, in unfair scenarios individuals often get angry and punish others. The study by Yu et al. (2015) has shown increased attentional resources allocated to receiving fair offers from an anonymous (versus known) person, reflected by the increased amplitude of P300. With regard to unfair scenarios, Wu et al. (2011) reported a less positive P300 compared to fair offers only in the anonymous condition. The authors conclude that contextual factors such as anonymity modulate fairness concerns. This may ultimately influence aggressive behavior as shown in previous studies assessing the effect of deindividuation on aggression (Lightdale and Prentice, 1994). However, such investigations are limited with regard to the localization of involved brain regions.

Potential regions for observing the effect of anonymity are those attributed to the TOM network. In the TAP, participants see how much money the opponent has subtracted, after which they have to choose the amount of money, they will subtract from the opponent. They know that the decision will only be implemented in case of the player winning the round. Thus, participants need to understand the intention and reason behind the opponent’s actions to select a corresponding reaction. However, in the anonymous scenario, participants do not have information regarding the opponent, and relevant details about the emotional state of the other (such as the opponent’s face) are absent. Missing information may exacerbate the interpretation of the other’s intention. Hence, it is expected that TOM network nodes will work differently during the anonymous interaction.

A primary hypothesis may be that TOM areas will be less involved when engaging with anonymous versus known people because less information about the opponent is present. Based on our assumptions, we expect a decrease in local activity in TOM-related structures or/and connectivity between them during interacting with an anonymous, compared to a known opponent. In line with this idea, performing an image-phrase compatibility task for images with blurred faces was characterized by decreased local activity in areas of visual social information processing (Proverbio et al., 2018). Furthermore, comparing the categorization of non-social scenes to the categorization of social scenes revealed reduced activity of the TOM areas: the bilateral temporal pole and superior temporal sulcus, mPFC, precuneus, and right IFG (Wagner et al., 2011).

An alternative hypothesis could be that hyper-involvement of the TOM areas may be required because the additional effort is applied to attribute thoughts, beliefs, and intentions to the anonymous opponent to understand his/her actions. Based on our assumption, we expect that increased local activity in TOM-related structures or/and connectivity between them should be found when comparing interaction with an anonymous opponent to interaction with a known one. Yu et al. (2015) have suggested a re-distribution of attentional resources in order to confirm the anonymous opponent’s identity. Similarly, conditions that involve a greater load on TOM-related social-cognitive processes on the neural level are characterized by increased activity of areas within the TOM system. Notably, compared to the task of predicting the intentions of another player (low-level TOM involvement), the task of predicting the thoughts of another player concerning one’s own intentions (high-level TOM involvement) during a strategic game has been associated with increased activity in the left anterior insula and right IFG (Bhatt and Camerer, 2005). In addition, the dorsolateral prefrontal cortex has been reported to encode the depth of reasoning about others’ thoughts (Yoshida et al., 2010), while the degree of how often people use high-level reasoning in strategic games correlated with activity in the medial prefrontal cortex (Coricelli and Nagel, 2009).

Finally, it might be the case that both increased and decreased activity or/and functional connectivity in distinct areas of the TOM network will be observed in association with anonymous interaction. Previous meta-analyses of effective connectivity studies on social cognition indicated that both negative coupling (i.e., segregation) and positive coupling (i.e., integration) between different networks do not imply a contradiction (Shine and Poldrack, 2018; Schurz et al., 2020a). Instead, this may reflect two rivaling constraints on cognitive function.

Based on the relevance of the TOM system during the evaluation of social contexts, such as provocation-aggression contexts, and the influence of social information provided about the other party, the objective of the current study was to study activity and connectivity of the TOM network while performing the TAP task against an anonymous or known opponent. We hypothesized that the local BOLD-signal and functional connectivity in the TOM areas reorganize and that this reorganization is twofold. On the one hand, a lack of information to process will be associated with decreased local activity and functional connectivity in some areas of the TOM system. On the other hand, as mentalizing during the interaction with an anonymous may be more demanding in terms of required resources, increased levels of local activity and distant interactions of other TOM system areas will be observed.

## Materials and Methods

### Participants

Forty-two healthy right-handed volunteers (26 females and 13 males, age 24.5 ± 3.6 years) without a history of psychiatric or neurological diseases or current medication intake were recruited via an advertisement placed on social network and took part in the experiment for a monetary reward (1,500 rubles). We assessed the handedness of the participants using the Edinburgh Handedness Inventory (Oldfield, 1971). All participants provided written informed consent prior to commencing the study. We performed all procedures in accordance with the Declaration of Helsinki, and they were approved by the Ethics Committee of the N.P. Bechtereva Institute of the Human Brain, St. Petersburg, Russia. After the experiment, the participants filled out a questionnaire concerning their game strategy and opinions about the opponent.

### Stimuli and Procedure

**FIGURE 1:**
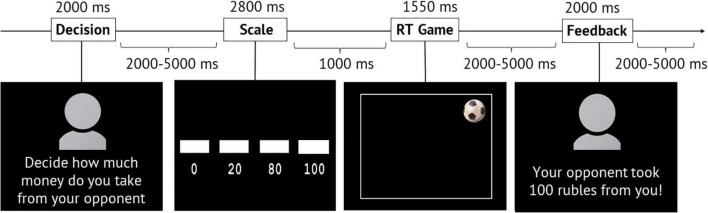
Structure and temporal characteristics of the modified TAP paradigm. During the “Scale” phase, a scale indicating the amount of money was gradually filled, and the volunteer had to press the button when the box appeared above the selected amount. RT, reaction time.

The participants were informed that they were going to play a reaction time game against two opponents. They became acquainted, spent some time with one of their opponents while receiving the instructions about the experiment, and learned that another opponent would remain anonymous before and after the experiment. The fMRI scanning consisted of four sessions presented in random order: In two of them, the volunteer played with a known opponent and in another two with an anonymous opponent. There were 160 trials across all four sessions: 80 in sessions with a known opponent and 80 in sessions with an anonymous opponent. Each trial (game round) consisted of four phases: “Decision,” “Scale,” “Game,” and “Feedback” (see Figure 1).

In the first phase (the “Decision” phase), the inscription “Decide how much money you will take from your opponent” and a picture—either with an anonymous avatar or a photo of a real opponent (whom they had recently met)—appeared on the screen. An avatar represented a silhouette, and no information (sex, age, appearance) could be inferred from it. According to the instructions, during this phase, the volunteers had to decide the amount of money they would subtract from the opponent in case of winning the round.

In the second phase (the “Scale” phase), a gradual filling scale indicating the sum of 0, 20, 80, and 100 roubles appeared on the screen. The volunteers had to press the button with their right index finger when the scale corresponded to the amount they chose earlier in the “Decision” phase.

In the third phase (the “Game” phase), an image of a soccer ball appeared randomly in one of the four corners of the playing field (screen). The task was to press the button with the right index finger as fast as possible upon seeing the ball. At the same time, the participants were asked not to press the button ahead of time (before the ball appeared).

In the fourth phase (the “Feedback” phase), either an avatar of an anonymous opponent or a photo of a real opponent appeared on the screen with the inscription indicating losing (“Your opponent took 0/20/80/100 roubles from you”!) or winning the game (“You won 50 roubles”!).

According to the instructions, the number chosen by the participant at the beginning of the round predisposed the amount of money lost by the opponent in case the volunteer won. The amount taken from the volunteers in case they lost depended, in turn, on their opponent’s choice. It was emphasized that the participants would not earn the money they took away from the opponent. The sum gained in case of victory was fixed to 50 roubles for all players. Thus, subtracting money from a known person or anonymous player could be defined as a reactive, aggressive action (Baron et al., 1994).

In reality, we programmed the “Feedback” phase: The participants won 60 trials across all four sessions, lost 0 and 100 roubles across 48 trials, and lost 20 and 80 roubles across 52 trials. These trials were presented in random order while being matched for the trails played against a known or anonymous opponent respectively. The programmed number of trials could only be changed if the volunteers did not press the button when catching the ball. In this case, 0 or 20 roubles were always taken from the volunteer during the “Feedback” phase. Observing the amount of money lost in the game was considered a social provocation, which could be high (80 or 100 roubles) or low (0 or 20 roubles).

The “Decision” phase lasted for 2 s. The duration of the “Scale” phase was 2.8 s (the first sum of the scale appeared for 1 s, three subsequent ones for 600 ms). On average, the “Game” phase lasted for 1.55 s (1.2–1.8 s), out of which for 700–1200 ms, no ball appeared on the screen, and for 600 ms, the ball was shown. Next, “Feedback” appeared on the screen for 2 s. The interval between the “Decision” and “Scale” phases varied from 2 to 5 s (average: 3.5 s). The interval between the “Scale” and “Game” phases was 1 s. The interval between the “Game” and “Feedback” phases varied from 2 to 5 s (average: 3.5 s). The interval between trials also varied from 2 to 5 s and averaged 3.5 s (see Figure 1).

### fMRI Image Acquisition Procedure and Image Processing

fMRI data were recorded using a 3 Tesla Philips Achieva scanner. Structural images were acquired prior to the task using a T1weighted pulse sequence (T1W3DFFE; repetition time [TR] = 25 ms; echo time [TE] = 2.2 ms; 30° flip angle), measuring 130 axial slices (field of view [FOV] = 240 × 240 mm) of 1 mm thickness and a 1 × 1 mm pixel size. Functional images were obtained using an echo-planar imaging (EPI) sequence (TR = 2 s, TE = 35 ms; 90° flip angle; FOV = 200 × 186 mm). In total, 32 continuous 3.5 mm thick axial slices (voxel size = 3 × 3 × 3.5 mm) covering the entire cerebrum and most of the cerebellum were oriented with respect to structural images.

An MR-compatible cervical collar was used to prevent head movements. Data preprocessing and subsequent statistical analyses were performed via SPM12 software^1^ run in MATLAB R2017a (Mathworks Inc., Natick, MA, United States, CШA). The preprocessing of raw fMRI data for each participant included the following stages: realignment, slice-time correction, co-registration, segmentation, normalization, and smoothing (8 mm FWHM). During the realignment stage, 6 parameters of head movement relative to the first image were generated (translations and rotations in three coordinate axes).

### Statistical Analysis

First, statistical analysis was performed for each participant separately, and individual general linear models (GLMs) were generated. The same GLM models, with 11 regressors representing the temporal characteristics of the experimental phases, were created for anonymous and known opponents. Events were classified similar to the GLMs created by Wagels et al. (2019), who also applied an fMRI compatible version of the TAP task. Namely, events were modeled with the onset at the beginning of the experimental phase and duration equal to zero. The “Feedback” phase (provocation) corresponded to three GLM regressors: low provocation (the loss of 0 or 20 roubles), high provocation (the loss of 80 or 100 roubles), and no provocation (winning). “Decision” and “Scale” phases were sorted according to provocation in the preceding trial: low, high, and no provocation. The “Game” phase corresponded to a separate regressor. GLMs also included first trials and mistakes in one separate regressor of no interest and six regressors for six head movement parameters obtained during preprocessing (realignment) (Johnstone et al., 2006). Regressors were then convolved with the standard hemodynamic response function (HRF).

Second, beta values of regression coefficients for the regressors in GLMs were estimated at the individual level of analysis. Linear contrasts of beta coefficients of each game phase and the baseline were calculated and used as a variable for the second-level analysis. At the second-level random-effect analysis, models were generated for each game phase of interest (“Decision,” “Scale,” and “Feedback”) separately and included two factors with two levels: “provocation” (high and low) and “opponent” (known and anonymous). F-contrasts for the main effect of the opponent, the main effect of the provocation, and the interaction between two factors were calculated.

Last, the obtained F-contrasts were used to make a voxelwise statistical inference on a group level. An uncorrected *p* < 0.001 threshold was applied at the voxel level, and a familywise error (FWE) corrected *p* < 0.05 threshold was applied at the cluster level. A gray matter mask, created from segmented structural images, was used to only select voxels within the gray matter in all subjects. Xjview Toolbox^2^ was used to identify the anatomical location of voxels, with significant changes in local neuronal activity. The REX toolbox was applied to illustrate differences in values of beta coefficients in obtained clusters of changes in BOLD signal^3^.

### Psychophysiological Interaction Analysis

To estimate the effect of anonymity on changes in functional connectivity, the generalized form of psychophysiological interactions analysis (gPPI-analysis) was performed using the toolbox for SPM12^4^ (McLaren et al., 2012). This method allows for distinguishing context-dependent changes in the strength of functional interactions from mere coactivations and correlations of spontaneous signal fluctuations observed during the resting state. ROI was selected based on two criteria. First, the ROI should be a node of the TOM network. Second, the ROI should be characterized by differential neuronal activity during the interaction with the known and anonymous opponent obtained in the current study. One cluster comprising the right IFG satisfied the named criteria as characterized by differential activity in our study and assigned to the TOM network in previous meta-analyses (Mar, 2011; Bzdok et al., 2012). In addition, the local BOLD signal in the right IFG was increased for the observation of provocation from the anonymous opponent compared to the known opponent: “Anonymous opponent > Known opponent” contrast calculated for the “Feedback” phase (see “*The effect of provocation on local brain activity changes during the observation of social provocation [the ‘Feedback’ phase]”* paragraph in the Results section). Thus, we selected the right IFG cluster, with the center at MNI coordinates *x* = 57, *y* = 11, *z* = 14, as the ROI in the gPPI analysis.

In the gPPI analysis, individual GLMs described above (see section “Statistical Analysis”) included additional regressors: a physiological regressor and PPI regressors. The physiological regressor *Xphysio(t)* represents the BOLD signal time series in the ROI. To create the PPI regressor *XPPI(t)*, BOLD signal time series from the ROI were deconvolved (⊗^^^-1) with HRF*(t)* to reveal underlying neuronal activity *Zphysio(t)*: *Zphysio(t)* = *Xphysio(t) ⊗^^^-1 HRF(t)* (Gitelman et al., 2003). The obtained signal was multiplied by the temporal characteristics of experimental events *Zpsy(t)*. The outcome of this multiplication represented the psychophysiological interaction on the level of neuronal activity. To model this interaction on a level of the BOLD signal, it was convolved with the HRF: *XPPI(t)* = *(Zphysio(t) ⋅ Zpsy(t)) ⊗ HRF(t).* PPI regressors were created separately for anonymous and known opponents and for regressors of interest, including the “Feedback” phase with low and high provocation. The analysis was performed for the “Feedback” phase because significant changes in local BOLD signals were registered for this phase of receiving provocation from the anonymous opponent.

Similar to the analysis of BOLD signal changes, the group-level model included two factors with two levels: “provocation” (high and low) and “opponent” (known and anonymous). F-contrasts for the main effect of the opponent, the main effect of the provocation, and the interaction between two factors were calculated.

An uncorrected *p* < 0.001 threshold was applied at the voxel level, and a FWE-corrected *p* < 0.05 threshold was applied at the cluster level. A gray matter mask, created from segmented structural images, was used to only select voxels within the gray matter in all subjects. xjView Toolbox^5^ (see footnote 2) was employed to identify the anatomical location of voxels, with significant changes in local neuronal activity. To interpret and illustrate results in terms of which clusters obtained in the whole-brain analysis are localized within the TOM neural system, thresholded maps of seven TOM-related regions (the right TPJ (rTPJ) and left TPJ; the precuneus; the dorsal, middle, and ventral components of the medial prefrontal cortex; and the right STS) were used (Dufour et al., 2013), and downloaded from https://saxelab.mit.edu/use-our-theory-mind-group-maps/. Results obtained in the current study were overlayed with the regions obtained by Dufour et al. (2013). Only those clusters that overlapped with TOM regions were interpreted to be localized within the TOM system.

REX toolbox (see footnote 3) was applied to illustrate differences in values of regression coefficients in obtained clusters of changes in functional interactions.

## Results

### Behavioral Results

In the statistical analysis, we included 39 out of 42 subjects (13 males/26 females, mean age 24.5 ± 3.6 years). We excluded three participants because their behavioral responses indicated that they did not believe or understand the instructions: One did not believe in having played against another human (according to the post-experimental questionnaire), one repeated the same order of answers throughout the experiment (no actual cognitive involvement), and one lost 74% of reaction time games due to pressing the button too fast (cheating).

When performing the TAP task, participants on average selected low punishment (subtracting 0 or 20 roubles) in 62% of the trials and high punishment (subtracting 80 or 100 roubles) in 38% of the trials. However, we did not detect any significant difference between the anonymous and known opponents in terms of the proportion of trials with low and high selected punishments.

### Imaging Results

#### The Effect of Anonymity on Local Brain Activity Changes During Decision-Making (the “Decision” Phase)

**FIGURE 2:**
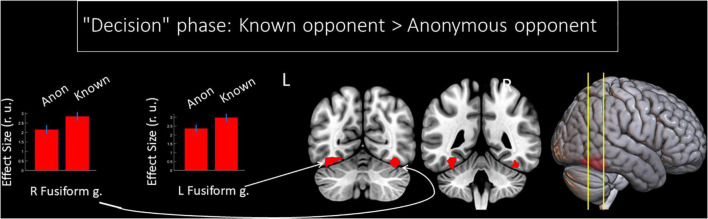
Results of the group-level BOLD signal analysis. Clusters of increased BOLD signals associated with decision-making after being provoked for the F-contrast “Known opponent vs. Anonymous opponent” (uncorrected *p* < 0.001 at the voxel level, FWE-corrected at the cluster level *p* < 0.05, *k* = 15). Plots show effect sizes with 95% confidence intervals. L/R, left/right hemisphere; g., gyrus; Anon, anonymous opponent; Known, introduced opponent.

We observed a main effect of the factor “opponent” on the BOLD signal changes during the phase of decision-making. This period comprised the time when the participant *thought about* the amount of money to subtract from the respective opponent after seeing his/her provocation. Compared to an anonymous person, the *thought about* reacting toward an introduced opponent was associated with increased BOLD signals in the fusiform gyrus bilaterally (see Figure 2 and Table 1). No voxels demonstrated a significant increase in the BOLD signal if participants *thought about* the amount to subtract from an anonymous opponent. We did not observe any significant changes for the main effect of the factor “provocation” (high or low) and the interaction of factors “opponent” and “provocation” for the “decision” phase.

**TABLE 1:** Results of the group-level analysis of the BOLD signal associated with decision-making after being provoked (uncorrected *p* < 0.001 on the voxel level, FWE-corrected at the cluster level *p* < 0.05, and *k* = 15).

	Cluster		Peak			Peak MNI coordinates		
Brain area	p(FWE-corr.)	k	p(unc.)	F	Z	x	y	z
***“Decision” phase: Known opponent* > *Anonymous opponent***								
L Fusiform g.	<0.001	160	<0.001	36.21	5.47	–36	–46	–19
R Fusiform g.	0.001	143	<0.001	34.71	5.37	39	–64	–16

***No significant changes were obtained for the “Decision” phase: Anonymous opponent* > *Known opponent***								

*k, cluster size in voxels; FWE, familywise error correction; L/R, left/right hemisphere; g., gyrus.*

#### The Effect of Provocation on Local Brain Activity Changes During the Selection of Punishment (the “Scale” Phase)

We observed a main effect of “provocation” during the Scale phase. *Subtracting* a high amount of money (80 or 100 roubles) compared to the low amount of money (0 or 20 roubles), irrespective of whether the opponent was introduced or not, was associated with an increased BOLD signal in the left putamen (see Table 2). No voxels demonstrated a significant increase in the BOLD signal during selection to *subtract* a low (versus high) amount of money. We did not observe any significant changes for the main effect of the factor “opponent” or the interaction of factors for the “Scale” phase.

**TABLE 2:** Results of the group-level analysis of the BOLD signal associated with selection of punishment (uncorrected *p* < 0.001 at the voxel level, FWE-corrected at the cluster level *p* < 0.05, and *k* = 15).

	Cluster		Peak			Peak MNI coordinates		
Brain area	p(FWE-corr.)	k	p(unc.)	F	Z	x	y	z
***“Scale” phase: High provocation* > *Low provocation***								
L Putamen	0.018	106	<0.001	26.49	4.73	–18	11	8

***No significant changes were obtained for the “Scale” phase: Low provocation* > *High provocation***								

*k, cluster size in voxels; FWE, familywise error correction; L/R, left/right hemisphere.*

#### The Effect of Anonymity on Local Brain Activity Changes During the Observation of Social Provocation (the “Feedback” Phase)

**FIGURE 3:**
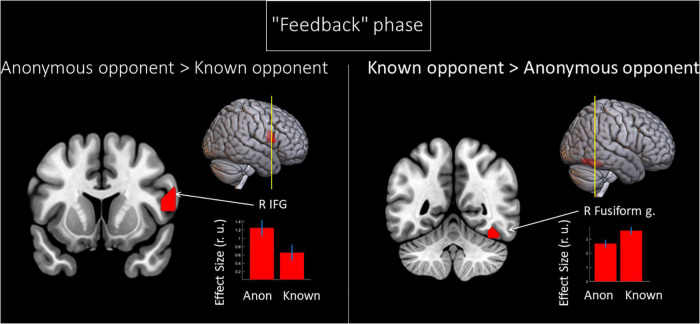
Results of the group-level BOLD signal analysis. Clusters of increased BOLD signals associated with the observation of provocation for the F-contrast “Anonymous opponent vs. Known opponent” (uncorrected *p* < 0.001 at the voxel level, FWE-corrected at the cluster level *p* < 0.05, and *k* = 15). Plots show effect sizes with 95% confidence intervals. L/R, left/right hemisphere; g., gyrus; IFG, inferior frontal gyrus; Anon, anonymous opponent; Known, introduced opponent.

We noted the main effect of the factor “opponent” on the BOLD signal changes during the phase when participants received the provocation (the “Feedback” phase). Compared to a known opponent, observing feedback from an anonymous opponent was associated with increased BOLD signals localized in the right IFG (see Figure 3 and Table 3). Compared to observing feedback from an anonymous person, receiving the provocation from a known opponent was associated with increased BOLD signals localized in the right fusiform gyrus (see Figure 3 and Table 3), resembling the finding for the “Decision” phase. We did not observe any effect of interaction between the two factors (“opponent” and “provocation”) for the “Feedback” phase.

**TABLE 3:** Results of the group-level analysis of the BOLD signal associated with the observation of provocation (uncorrected *p* < 0.001 at the voxel level, FWE corrected at the cluster level *p* < 0.05, and *k* = 15).

	Cluster		Peak			Peak MNI Coordinates		
Brain area	p(FWE-corr.)	k	p(unc.)	F	Z	x	y	z
***“Feedback” phase: Anonymous opponent* > *Known opponent***								
R Inferior frontal g.	0.009	111	<0.001	29.65	4.99	57	11	14

***“Feedback” phase: Known opponent* > *Anonymous opponent***								
R Fusiform g.	0.005	126	<0.001	58.47	6.76	39	–52	–19

*k, cluster size in voxels; FWE, familywise error correction; L/R, left/right hemisphere; g., gyrus.*

#### The Effect of Provocation on Local Brain Activity Changes During the Observation of Social Provocation (the “Feedback” Phase)

When observing provocative feedback from opponents, high provocation (80 or 100 roubles), compared to low provocation (0 or 20) roubles, was associated with an increased local BOLD signal in the angular gyrus and supplementary motor area (see Table 4). Compared to high provocation, low provocation was associated with increased BOLD signals localized in the right middle frontal gyrus and left IFG, the left angular gyrus, and the right precuneus (see Table 4). Among these structures, the right angular gyrus, left angular gyrus, and right precuneus are localized within the rTPJ, left TPJ, and precuneus clusters of the TOM system, respectively, according to masks generated by Dufour et al. (2013). We did not observe any effect of interaction between the two factors (“opponent” and “provocation”) for the “Feedback” phase.

**TABLE 4:** Results of the group-level analysis of the BOLD signal associated with the observation of provocation (uncorrected *p* < 0.001 at the voxel level, FWE-corrected at the cluster level *p* < 0.05, and *k* = 15).

	Cluster		Peak			Peak MNI coordinates		
Brain area	p(FWE-corr.)	k	p(unc.)	F	Z	x	y	z
***“Feedback” phase: High provocation* > *Low provocation***								
R Angular g.	<0.001	502	<0.001	48.33	6.23	48	–52	29
R SMA	0.001	173	<0.001	40.66	5.77	6	11	62

***“Feedback” phase: Low provocation* > *High provocation***								
R Middle frontal g.	<0.001	749	<0.001	48.12	6.21	39	20	44
L Angular g.	0.001	166	<0.001	29.15	4.95	–54	–58	38
L Inferior frontal g.	0.001	176	<0.001	24.95	4.60	–39	20	32
R Precuneus	0.002	151	<0.001	24.84	4.59	6	–55	41

*k, cluster size in voxels; FWE, familywise error correction; L/R, left/right hemisphere; g., gyrus; SMA, supplementary motor area.*

#### Functional Connectivity Changes of the Right Inferior Frontal Gyrus: The Effect of Anonymity on the Observation of Social Provocation During the “Feedback” Phase

**FIGURE 4:**
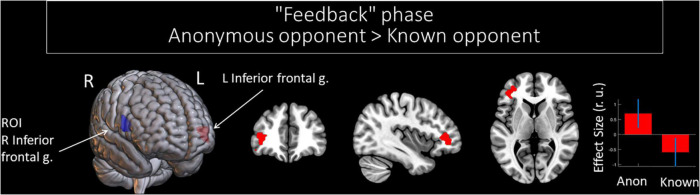
Results of the group-level gPPI analysis with the ROI in the right IFG. Clusters of significant changes in functional connectivity, with the right IFG associated with the anonymity of the opponent (uncorrected *p* < 0.001 at the voxel level, FWE-corrected at the cluster level *p* < 0.05, and *k* = 15), are shown in red. The ROI in the right IFG is presented in blue. The plot shows effect sizes with 95% confidence intervals. L/R, left/right hemisphere; g., gyrus; ROI, the region of interest; Anon, anonymous opponent; Known, introduced opponent.

Compared to a known opponent, observing feedback from an anonymous opponent was associated with increased functional connectivity between the right and left IFGs (see Figure 4 and Table 5). However, we did not witness any significant changes in the functional connectivity of the right IFG for the observed provocation from the known opponent compared to the anonymous opponent.

**TABLE 5:** Results of the group-level gPPI analysis with the ROI in the right IFG, associated with the observation of provocation from anonymous and known opponents (uncorrected *p* < 0.001 at the voxel level, FWE-corrected at the cluster level *p* < 0.05, and *k* = 15).

	Cluster		Peak			Peak MNI coordinates		
Brain area	p(FWE-corr.)	K	p(unc.)	F	Z	x	y	z
***“Feedback” phase: Anonymous opponent* > *Known opponent***								
L Inferior frontal g.	0.011	91	<0.001	22.20	4.34	–39	41	5

***No significant changes were obtained for the “Feedback” phase: Known opponent* > *Anonymous opponent***								

*k, cluster size in voxels; FWE, familywise error correction; ROI, region of interest; L/R, left/right hemisphere; g., gyrus.*

#### Functional Connectivity Changes of the Right Inferior Frontal Gyrus: The Effect of the Level of Provocation on the Observation of Social Provocation During the “Feedback” Phase

When observing provocative feedback from opponents, low provocation (0 or 20 roubles), compared to high provocation (80 or 100 roubles), irrespective of whether the opponent was introduced or not, was associated with increased functional connectivity of the right IFG with the right superior temporal gyrus and left cerebellum (see Table 6). However, no significant changes in the functional connectivity of the right IFG were associated with observing high provocative feedback compared to low provocative feedback (0 or 20 roubles) from opponents.

**TABLE 6:** Results of the group-level gPPI analysis with the ROI in the right IFG, associated with the observation of different levels of provocation (uncorrected *p* < 0.001 at the voxel level, FWE-corrected at the cluster level *p* < 0.05, and *k* = 15).

	Cluster		Peak			Peak MNI coordinates		
Brain area	p(FWE-corr)	K	p(unc)	F	Z	x	y	z
***“Feedback” phase: Low provocation* > *High provocation***								
R Superior temporal g.	0.054	59	<0.001	22.87	4.41	51	–10	–4
L Cerebellum	0.002	133	<0.001	20.36	4.16	–6	–49	–4

***No significant changes were obtained for the “Feedback” phase: High provocation* > *Low provocation***								

*k, cluster size in voxels; FWE, familywise error correction; ROI, region of interest; L/R, left/right hemisphere; g., gyrus.*

#### Functional Connectivity Changes of the Right Inferior Frontal Gyrus: Interactions Between Anonymity and the Level of Observed Provocation Regarding the Observation of Social Provocation During the “Feedback” Phase

**FIGURE 5:**
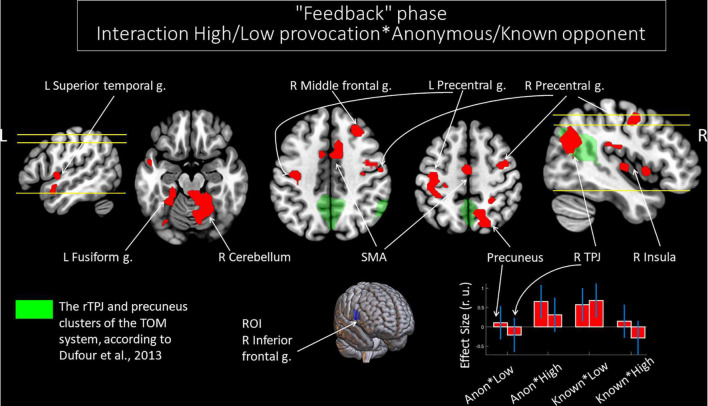
Results of the group-level gPPI analysis with the ROI in the right IFG. Clusters of significant changes in functional connectivity with the right IFG, associated with the interactions between the level of observed provocation and the anonymity of the opponent (uncorrected *p* < 0.001 at the voxel level, FWE-corrected at the cluster level *p* < 0.05, and *k* = 15), are shown in red. The TOM system clusters are presented in green according to the results of Dufour et al. (2013). The plot on the lower right side indicates effect sizes with 95% confidence intervals. L/R, left/right hemisphere; g., gyrus; TPJ, temporoparietal junction; SMA, supplementary motor area; ROI, region of interest; Anon, anonymous opponent; Known, introduced opponent; High, high provocation; Low, low provocation.

We noted significant changes in functional connectivity for the ROI in the right IFG and the interactions between the factors of “opponent” and “provocation.” The right IFG changes functional connectivity with the bilateral cerebellum, precentral gyrus, and supplementary motor area; left fusiform gyrus and superior temporal gyrus; right middle frontal gyrus, insula, precuneus, and angular gyrus (see Table 7). Among these structures, the right angular gyrus and right precuneus are localized within the rTPJ and precuneus clusters of the TOM system, respectively, according to masks generated by Dufour et al. (2013) (see Figure 5). During the perception of the provocation from the anonymous opponent, functional interactions between the IFG and the named areas were increased in the condition of high (versus low provocation). Along with that, this interaction was inversed compared to observing provocation from the known opponent. We did not observe any significant results for other directions of comparison.

**TABLE 7:** Results of the group-level gPPI analysis with the ROI in the right IFG for the interaction between the level of observed provocation and the anonymity of the opponent during the “Feedback” phase (uncorrected *p* < 0.001 at the voxel level, FWE-corrected at the cluster level *p* < 0.05, and *k* = 15).

	Cluster		Peak			Peak MNI coordinates		
Brain area	p(FWE-corr.)	k	p(unc.)	F	Z	x	y	z
R cerebellum	<0.001	457	<0.001	57.83	6.72	9	–37	–22
L cerebellum	0.019	80	<0.001	48.98	6.26	–30	–58	–34
R angular g.	0.001	150	<0.001	48.16	6.22	42	–61	29
R middle frontal g.	0.054	59	<0.001	45.84	6.08	30	32	44
L precentral g.	<0.001	195	<0.001	38.10	5.60	–36	–16	50
R insula	<0.001	363	<0.001	37.96	5.59	36	8	2
R SMA	0.010	95	<0.001	36.68	5.51	12	8	47
L fusiform g.	0.041	64	<0.001	26.57	4.74	–30	–49	–16
L superior temporal g.	<0.001	232	<0.001	26.46	4.73	-51	2	–13
R precuneus	<0.001	190	<0.001	25.84	4.68	12	–58	53
R precentral g.	0.020	79	<0.001	24.47	4.55	39	–4	50
L SMA	0.002	127	<0.001	20.80	4.21	0	–10	56

*During the perception of the provocation from the anonymous opponent, functional interactions between the IFG and the listed areas were increased in the condition of high (versus low provocation). This interaction was inversed compared to observing provocation from the known opponent. We did not observe any significant results for other directions of comparison.*

*k, cluster size in voxels; FWE, familywise error correction; ROI, region of interest; L/R, left/right hemisphere; g., gyrus; SMA, supplementary motor area.*

## Discussion

The current fMRI study demonstrates differences in both activity and functional connectivity within the TOM network associated with observing anonymous versus known opponent provocations during a modified TAP task. This paradigm allows studying only competitive scenarios while social interactions presume cooperation as well (Decety et al., 2004; Kanske et al., 2015a; Tsoi et al., 2016; Liu et al., 2017; Bitsch et al., 2018). Although participants can decide not to compete, in the TAP there is no possibility to cooperate which may result in very different interactions. Therefore, the obtained data should be attributed only to competitive interactions. While we did not witness any behavioral differences in terms of punishment selected by our subjects for different opponents, the data support the hypothesis about the increased involvement of TOM network nodes during the evaluation of anonymous opponent’s actions in contrast to known opponent’s actions. Compared to observing provocation from a known opponent, being provoked by an anonymous opponent was underpinned by an increased BOLD signal in the right IFG and increased functional connectivity between the right and left IFGs. In addition, we noted increased functional connectivity between the right IFG, rTPJ, and precuneus, when observing high provocation (subtraction of a high amount of money) from an anonymous opponent versus a known opponent.

The obtained results indicate that functional connectivity in the TOM network changes depending on the knowledge an opponent. In more detail, higher functional connectivity seems to support interactions with anonymous in contrast to known opponents. We speculate that these changes underlie the compensatory process of “completing” socially relevant details, as if by “thinking out” this missing information. Furthermore, only specific parts of the TOM network demonstrated increased involvement in the interaction with the anonymous vs. known opponent. This may generate the hypothesis for a twofold character of re-organization of the TOM network: although the bilateral IFG, rTPJ, and precuneus are the nodes in the TOM network (Mar, 2011; Bzdok et al., 2012; Dufour et al., 2013; Molenberghs et al., 2016), the results do not include the exhaustive list of other network nodes. Our results are in line with the fact that the TOM network is heterogeneous. Indeed its nodes have been shown to demonstrate differential involvement in performance, including the activity and interaction between different underlying resting-state networks (Default Mode and Control networks), depending on the experimental task used (Molenberghs et al., 2016; Schurz et al., 2020a). Thus, obtained results in the current study support the assumption that social interactions with an anonymous opponent in a competitive context are associated with re-organization of the TOM network functioning. The observed re-organization consists of hyperactivation and increased functional connectivity in specific parts of the TOM network. A hypothesis to be systematically tested in future studies may be that the re-organization is needed to compensate for the lack of socially relevant information.

### Increased Activity in the Right Inferior Frontal Gyrus and Increased Functional Connectivity Between the Right and Left Inferior Frontal Gyrus in the Anonymous Condition Underlie the Compensatory Visual Face Processing Mechanism

In addition to being part of the TOM network, the bilateral IFG is a key node in the mirror neuron system (Rizzolatti and Craighero, 2004; Cattaneo and Rizzolatti, 2009). This attribution is supported by meta-analysis (Molenberghs et al., 2012). It is generally assumed that the mirror neuron system is responsible for understanding the goals and intentions of others’ motor acts by matching them to one’s own motor repertoire (Rizzolatti et al., 2001; Rizzolatti and Fabbri-Destro, 2008).

In the same vein, areas of the mirror neuron system are involved in observing facial expressions. The face conveys information about an individual’s emotional state, and allows the observer to access the individual’s emotional status (Ferrari and Coudé, 2018). For example, the gray matter volume in the right IFG is associated with the ability to recognize emotions via other people’s facial expressions (Uono et al., 2017). In addition, the stimulation of this area increases performance in facial emotion and identity perception tasks (Penton et al., 2017). Moreover, increased activity in the right IFG was observed when, by viewing photographs of faces, participants assessed the likelihood that a society or the participant himself/herself would interact with the presented person (Yamakawa et al., 2009). Meta-analyses also confirm higher activation associated with emotional face vs. neutral face contrast in the right IFG (Liu et al., 2021), left IFG (Schirmer, 2018), and IFG in both hemispheres (Sabatinelli et al., 2011; Müller et al., 2018).

This functional role of the bilateral IFG may be of particular importance for processing provocation. For example, when receiving feedback in the present study, the participants saw both the provocative inscription and the picture of their opponent, who was giving the feedback: either an avatar (a silhouette with no information about the true appearance) of an anonymous opponent or a photo of a real opponent appeared on the screen (see Figure 1). Thus, information about the face of the anonymous opponent was absent. While this is speculative at this stage, the interaction with the anonymous opponent could have required the compensatory, resource-demanding process induced by the lack of socially relevant face information. This is reflected by areas associated with face processing: some of them demonstrate increased involvement (right IFG activity and functional connectivity with the left IFG), while others underperform. The latter is valid for the right fusiform gyrus associated with the decreased level of the BOLD signal for the anonymous opponent compared to the known opponent, while receiving punishment after losing the game, irrespective of the selected punishment level.

### Potential Mechanisms Underpinned by the Increased Functional Connectivity of the Right Inferior Frontal Gyrus With the rTPJ and Precuneus in the Anonymous Condition

With regard to functional connectivity, the rTPJ and precuneus showed increased connectivity with the right IFG during the observation of high provocation in the anonymous condition compared to the known condition.

One possible mechanism that could explain this outcome are the interactions between different components of the TOM ability. A recent meta-analytic study characterized a number of TOM subcomponents and allocated social neurocognitive processes into three groups: (1) predominantly cognitive processes; (2) more affective processes; (3) combined processes, which engage cognitive and affective functions in parallel (Schurz et al., 2020b). The affective TOM component refers to the capacity to represent valence, emotions, and feelings, whereas the cognitive TOM component concerns valence-free, unemotional inference of others’ mental states (Brothers and Ring, 1992). Meta-analyses performed for cognitive and affective TOM tasks separately demonstrated that tasks requiring affective TOM showed increased involvement of the bilateral IFG, whereas the bilateral TPJ and precuneus were attributed to the cognitive aspect of TOM (Schurz et al., 2014; Molenberghs et al., 2016). In line with that, clustering meta-analyses have found activation changes in temporoparietal areas related to the cognitive component and signal alterations across the right frontal cortex, peaking in the IFG, related to the affective component (Schurz et al., 2020b).

Contrary to these findings, increased BOLD signals in the rTPJ and precuneus were also associated with the affective TOM condition (Bodden et al., 2013), while involvement of the right IFG underpinned cognitive perspective taking (Hynes et al., 2006) in direct comparisons of the affective (versus cognitive) TOM. In addition, affective and cognitive TOM conditions had common activations in the bilateral TPJ (Sebastian et al., 2012; Corradi-Dell’Acqua et al., 2014). In the work of Kanske et al. (2015a,b), similar results were obtained: two neighboring but distinct peaks related, one to affect sharing, and another to understanding others’ mental states, were detected in the temporoparietal cortex. This indicated that dorsal and ventral subregions of the TPJ are involved in different aspects of social-cognitive mechanisms.

Despite this distinction, previous studies highlight the integration between named areas. For example, the right IFG was one of the regions with the highest level of overlap across PPI analyses for all social brain regions (including the bilateral TPJ and precuneus) during a social evaluation task, in which participants were asked to consider others’ thoughts about themselves (McCormick et al., 2018). It was also argued that affective and cognitive routes of understanding others may coactivate and cooperate in complex social situations (Kanske, 2018; Schurz et al., 2020b). Specifically, Schurz et al. (2020b) have found that clusters in the bilateral IFG, attributed to tasks, requiring affective TOM, and clusters in the bilateral TPJ, and precuneus, attributed to tasks, requiring cognitive TOM, overlap with neural activations associated with the third cluster of tasks (intermediate). The third cluster comprises tasks which engage cognitive and affective functions in parallel. From this angle, the observed connectivity between these regions may reflect the increased requirements of both TOM components and their interactions. The involvement of different TOM components may be needed to figure out the reasons or motives as well as potential emotional states related to the high provocation from the anonymous (vs. known) opponent when the clear socially relevant information was lacking.

Taken together, these studies suggest that the additional involvement of the rTPJ and precuneus, through increased functional connectivity, characterizes the requirement of different aspects of TOM ability or higher-order analysis of social information as a compensatory mechanism during processing anonymous provocation. Since no systematic behavioral differences in the interactions with a known or anonymous opponent were observed, we assume that the anonymous status itself is associated with the connectivity changes. One possible explanation would be that the increased connectivity reflects the attempt to understand the opponent by “completing” socially relevant details.

### Limitations and Suggestions for Further Research

The main limitation of the current study is connected to the experimental design used. The effect of anonymity was calculated in the context of competitive social interaction using the TAP task. Although this task is widely used in psychophysiological studies of aggression, it is discussed controversially, how aggression should be defined using the TAP (Elson et al., 2014; McCarthy and Elson, 2018). Namely, participants’ motives for subtracting money from the opponent cannot be unambiguously measured and linked to prior provocations from the opponent. Furthermore, larger amounts of subtracted money do not obligatory reflect only higher levels of aggression. Even though aggression is not the main concern of the current study, the limitation is related to the classification of experimental events. Also, a reaction to provocation may significantly differ depending on individual differences between participants (Hyatt et al., 2019). Collecting and considering psychometric data may further characterize the obtained results and demonstrate otherwise undiscovered effects of anonymity in different groups of subjects. Notably, anonymous interactions occur in different social settings not limited to competitive games as applied in this experiment. Therefore, future research is required to clarify, if the observed effects are fundamental and can be applied to other conditions of social interaction.

## Conclusion

For the current study, we modified the TAP by introducing to this paradigm an anonymous opponent to examine the reorganization of the TOM brain system in settings of deficits with socially relevant information. A competitive interaction with an anonymous (compared to known) person was associated with functional reorganization in the TOM network: both functional activity and functional connectivity of and between several network nodes were increased. Due to no systematic behavioral differences in the interaction with a known or anonymous opponent, these activity and connectivity changes refer to the degree of knowledge about the opponent. We speculate that the neural changes may underlie different inferences about the opponents’ mental states. The idea that this reorganization of the TOM network reflects the attempt to understand the opponent by “completing” socially relevant details requires further investigation. The obtained data extend the current view on how the brain processes socially relevant information.

## Data Availability Statement

The raw data supporting the conclusions of this article will be made available by the authors, without undue reservation.

## Ethics Statement

The studies involving human participants were reviewed and approved by Ethics Committee of the N.P. Bechtereva Institute of the Human Brain, St. Petersburg, Russia. The patients/participants provided their written informed consent to participate in this study.

## Author Contributions

MV, RM, AK, and MK conceived and designed the analysis. AM, RM, and MZ collected the fMRI data. RM, AM, MK, and MV performed the analysis of fMRI data. MZ, AM, MV, DC, UH, LW, MD, MK, and AK wrote the manuscript. All the authors reviewed the manuscript and approved the submitted version.

## Conflict of Interest

The authors declare that the research was conducted in the absence of any commercial or financial relationships that could be construed as a potential conflict of interest.

## Publisher’s Note

All claims expressed in this article are solely those of the authors and do not necessarily represent those of their affiliated organizations, or those of the publisher, the editors and the reviewers. Any product that may be evaluated in this article, or claim that may be made by its manufacturer, is not guaranteed or endorsed by the publisher.

## Footnotes

1
http://www.fil.ion.ucl.ac.uk/spm

2
http://www.alivelearn.net/xjview

3
http://www.nitrc.org/projects/rex/

4
http://www.nitrc.org/projects/gppi
